# Involvement of lncRNAs in the tumor microenvironment: a new property of tumor immunity

**DOI:** 10.20892/j.issn.2095-3941.2023.0163

**Published:** 2023-08-26

**Authors:** Qingyu Lin, Jiaqi Zhu, Ying Hu

**Affiliations:** 1 School of Life Science and Technology, Harbin Institute of Technology, Harbin 150001, China; 2 Zhengzhou Research Institute of Harbin Institute of Technology, Zhengzhou 450000, China; 3 Key Laboratory of Science and Engineering for the Multi-modal Prevention and Control of Major Chronic Diseases, Ministry of Industry and Information Technology, Zhengzhou 450000, China; 4 School of Astronautics, Harbin Institute of Technology, Harbin 150001, China

Immune checkpoint blockade (ICB) has achieved durable clinical responses and has significantly improved the overall survival of cancer patients^1^. Among the ICB agents, programmed death 1 (PD-1)/programmed death ligand 1 (PD-L1) antibodies are used to treat various human tumors by blocking PD-1/PD-L1 signaling. Impressive response rates with low autoimmune toxicity have been reported in 20%–30% of non-selected patients^1^; however, most patients fail to respond to PD-1/PD-L1 blockade or acquire resistance during therapy through unknown mechanisms^1^. Recently, clinical data have indicated that PD-L1 expression and tumor-infiltrating lymphocyte density are associated with a better response to anti-PD-1/PD-L1 therapy^1^. Indeed, these findings highlight the urgent need for a better understanding of the molecular mechanisms underlying PD-L1 regulation because such knowledge may facilitate the development of alternative ICB strategies or the design of more precise and effective combinations of immune checkpoint therapies. In addition, dysregulated immune cells residing in the tumor microenvironment (TME) may contribute to increased cancer immune tolerance. Moreover, the intrinsic mechanisms involved in regulating immune cell function may be potential targets for switching from an immunosuppressive to an active status.

Long non-coding RNAs (lncRNAs) are a class of transcripts ≤ 200 nucleotides in length with no coding potential^2^. LncRNAs are multifunctional molecules that interact with RNA, DNA, or proteins to influence key signaling pathways in different cellular contexts, and thus lncRNAs engage in numerous physiologic and pathologic processes, including processed involving tumors^2^. Increasing evidence has also demonstrated the functional relevance of lncRNAs in directly controlling PD-L1 expression and regulating immune cell activities within the TME to affect the clinical outcome of PD-1/PD-L1 blockade^3,4^.

## Regulation of the PD-1/PD-L1 pathway in cancer by lncRNAs

**Figure 1:**
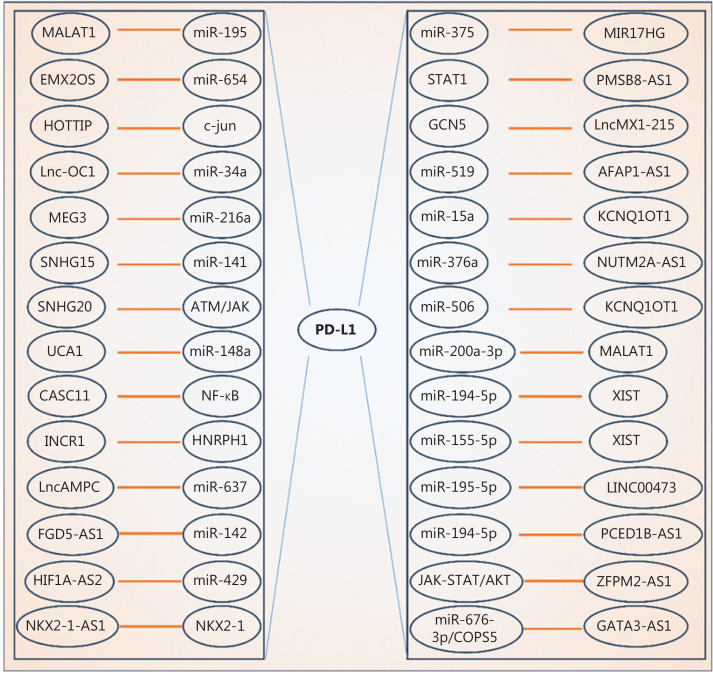
LncRNAs regulate PD-L1 expression. LncRNAs are involved in controlling *PD-L1* expression by interacting with different miRNAs and factors or by regulating various signaling pathways.

PD-L1 has been shown to be a biomarker for patient prognosis or tumor immunotherapy success^5^. Aberrant PD-L1 expression is frequently detected in various human cancers, so significant efforts have been made to understand how PD-L1 is regulated in cancer cells. PD-L1 levels are subjected to diverse regulatory mechanisms at the genetic, epigenetic, and post-translational levels^6^. Previous studies have provided additional markers to predict ICB efficacy, strategies to improve PD-L1 detection in tumor samples, and avenues to modulate PD-L1 activity. More recently, PD-L1 regulation by lncRNAs has been reported (**Table 1**, **Figure 1**). Notably, most of these lncRNAs, including *LINC00473*, *FGD5*-*AS1*, and *MALAT1*, regulate PD-L1 expression primarily *via* sponging microRNAs (miRNAs)^7,8^. LncRNAs localize to the nucleus, cytoplasm, or exosomes, and thus are able to regulate gene expression in many different ways. For example, *NKX2-1-AS1*, *ZFPM2*-*AS1*, and *lncMX1*-*215* regulate tumor immunity by mediating PD-L1 expression binding to different partners^7,8^. Different lncRNAs have been shown to regulate PD-L1 expression at different levels^3^; however, most of these studies have focused on miRNA sponging function of lncRNAs. Until recently, regulation of PD-L1 expression mediated by lncRNAs has been reported to occur at the translational level, independent of miRNAs.

**Table 1:** Overview the roles of lncRNAs in regulating PD-L1 expression

LncRNA	Cancer types	Regulated targets	Mechanisms	Reference
MALAT1	DLBCL	miR-195	MALAT1 sponges miR-195 to promote PD-L1 expression	^7^
EMX2OS	OC	miR-654	EMX2OS interacts with miR-654 to increase PD-L1 expression	^7^
MIR17HG	CRC	miR-375	MIR17HG induces PD-L1 expression *via* sponging miR-375	^7^
GATA3-AS1	BC	miR-676-3p/COPS5	GATA3-AS1 increases PD-L1 expression by regulating the miR-676-3p/COPS5 pathway	^7^
KCNQ1OT1	PC	miR-15a	KCNQ1OT1 activates PD-L1 expression through binding to and inactivating miR-15a	^7^
MALAT1	LAD	miR-200a-3p	MALAT1 increases PD-L1 expression by sponging miR-200a-3p	^7^
FGD5-AS1	LAD	miR-142	LncRNA FGD5-AS1 acts as a sponge of miR-142 to regulate PD-L1 expression	^7^
LINC00473	PC	miR-195-5p	LINC00473 enhances PD-L1 expression by binding to miR-195-5p	^7^
KCNQ1OT1	HCC	miR-506	KCNQ1OT1 interacts with miR-506 to promote PD-L1 expression	^7^
PCED1B-AS1	HCC	miR-194-5p	PCED1B-AS1 enhances PD-L1 expression through binding to miR-194-5p	^7^
Lnc-OC1	EC	miR-34a	Lnc-OC1 enhances PD-L1 expression by sponging miR-34a	^7^
MEG3	EC	miR-216a	MEG3 upregulates PD-L1 expression by targeting miR-216a	^7^
SNHG15	GC	miR-141	SNHG15 increases PD-L1 expression by targeting miR-141	^7^
HIF1A-AS2	GC	miR-429	HIF1A-AS2 contributes PD-L1 expression through binding to miR-429	^7^
NUTM2A-AS1	GC	miR-376a	NUTM2A-AS1 enhances PD-L1 expression by sponging miR-376a	^7^
CASC11	HCC	NF-κB	CASC11 activates NF-κB to further increase PD-L1 expression	^8^
XIST	HCC	miR-194-5p/miR-155-5p	XIST promotes PD-L1 expression *via* targeting miR-194-5p and miR-155-5p	^8^
PMSB8-AS1	PC	STAT1	PMSB8-AS1 increases STAT1 expression to further promote PD-L1 expression	^8^
NKX2-1-AS1	LAD	NKX2-1	NKX2-1-AS1 inhibits PD-L1 expression through modulating NKX2-1 expression	^8^
ZFPM2-AS1	LAD	JAK-STAT/AKT	ZFPM2-AS1 increases PD-L1 expression *via* regulating JAK-STAT/AKT pathways	^8^
LncAMPC	PC	miR-637	LncAMPC acts as a sponge of miR-637 to regulate PD-L1 expression	^8^
INCR1	Thy	HNRNPH1	Binding between INCR1 and HNRNPH1 suppresses PD-L1 expression	^8^
LncMX1-215	HNSCC	GCN5	LncMX1-215 negatively regulates PD-L1 expression by suppressing H3K27 acetylation *via* binding to GCN5	^8^
AFAP1-AS1	NPC	miR-519	AFAP1-AS1 upregulates PD-L1 expression *via* directly targeting miR-519	^8^
SNHG20	ESCC	ATM/JAK	SNHG20 regulates PD-L1 expression through modulation of the ATM/JAK pathway	^8^
UCA1	ATC	miR-148a	UCA1 interacts with miR-148a to promote PD-L1 expression	^8^

DLBCL, diffuse large B cell lymphoma; OC, ovarian cancer; EC, endometrial cancer; GC, gastric cancer; HCC, hepatocellular carcinoma; PC, pancreatic cancer; LAD, lung adenocarcinoma; BC, breast cancer; Thy, thymoma; HNSCC, head and neck squamous cell carcinoma; NPC, nasopharyngeal carcinoma; ESCC, esophageal squamous cell carcinoma; ATC, anaplastic thyroid carcinoma; CRC, colorectal cancer.

The correlation between lncRNAs and PD-L1 expression highlights the predictive and targetable value of lncRNAs in the response to and efficacy of PD-1/PD-L1 blockade immunotherapy; however, lncRNAs possess cell type-specific features, with relatively low levels of expression. To date, most lncRNA studies have relied heavily on deep RNA sequencing, in which various cell types contribute to an average signal, limiting the discovery of cell type-specific lncRNA functions^9^ because PD-L1 is expressed in different cell types, including cancer cells, immune-related cells, endothelial cells, and fibroblasts. PD-L1 expression, particularly in cancer and immune-related cells, is closely associated with ICB efficacy. Single-cell RNA-sequencing is a potential solution to overcome these limitations, despite the lack of annotations for low abundance, yet cell type-specific lncRNAs^9^. Furthermore, combining several lncRNAs with PD-L1 for prediction and targeting may be more conducive to benefit anti-PD-1/PD-L1 immunotherapy.

## LncRNAs regulate innate immune cells in the tumor immune microenvironment (TIME)

**Figure 2:**
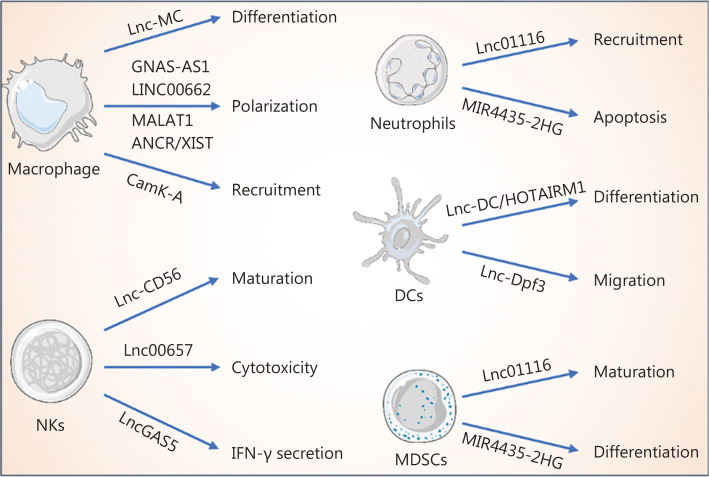
LncRNAs regulate innate immune cells. LncRNAs mediate macrophage differentiation and regulate macrophage polarization and recruitment. In addition, the recruitment and apoptosis of neutrophils are regulated by lncRNAs. Furthermore, lncRNAs mediate the maturation, cytotoxicity, and IFN-γ secretion of NKs. In addition to the regulatory functions in innate immune cells described above, lncRNAs control dendritic cell differentiation, migration, and MDSC maturation and differentiation.

One of the main goals of immune checkpoint inhibitors is to boost effector T cell activity. A positive correlation between T cell infiltration and effector T cell activity in the TME has been demonstrated; however, T cells are not autonomous with respect to anti-tumor functions. The triggering and maintenance of anti-tumor T cell responses depend on innate immune responses^10^. The innate immune system includes various cell types, such as myeloid lineage cells, natural killer cells (NKs), and dendritic cells (DCs)^10^. When a tumor is recognized by innate immune cells, these cells launch an adaptive immune response that results in tumor regression mediated by the killing effect of cytotoxic T cells. LncRNAs have recently been reported to be associated with the differentiation, polarization, recruitment, apoptosis, maturation, and cytotoxicity of innate immune cells by regulating functional gene expression^11^ (**Table 2**, **Figure 2**). Neutrophils derived from myeloid progenitor cells participate in innate and adaptive responses. Different polarization states have contradictory effects on anti-tumor immune responses. N1-type neutrophils kill tumor cells *via* antibody-dependent cell-mediated cytotoxicity after the initiation of an adaptive response, while N2-type cells have been reported to inhibit T cell activation by inducing arginase 1 (ARG1) and ROS. It has been demonstrated that lncRNAs, such as *Lnc01116* and Mir44*53-2HG*, participate in neutrophil recruitment and apoptosis^11,12^. Macrophages are phagocytic cells that are critical effector cells in innate immunity. In addition to phagocytic activity, macrophages contribute to the initiation of adaptive immune responses by releasing cytokines and chemokines. Macrophage types include M1 and M2 phenotypes. M1-type macrophages suppress tumor growth, whereas M2-type macrophages promote tumor progression. Several studies have shown that lncRNAs, such as *Lnc-MC*, *GNAS-AS1*, *LINC00662*, *MALAT1*, *ANCR*, *XIST*, and *CamK-A*, have important roles in macrophage differentiation, polarization, and recruitment^11^. NKs are innate lymphoid cells with cytotoxic effector functions. These cells are characteristically efficient at killing malignant cells with no MHC restrictions and at limiting tumor progression. *Lnc-CD56*, *Lnc00657*, and *LncGAS5* have been reported to regulate the maturation and cytotoxicity of NKs^11^. DCs are professional antigen-presenting cells and are responsible for recognizing danger-associated molecular patterns (DAMPs) or pathogen-associated molecular patterns (PAMPs) to initiate specific T cell responses and are therefore important in promoting protective immunity. Within the cancer framework, DCs carry tumor antigens, which are presented to T cells to activate anti-tumor responses. The antigen-presenting activity of DCs is mediated by maturation and migration capacities. *Lnc-DC*, *HOTAIRM1*, and *Lnc-Dpf3* have been reported to participate in the differentiation and migration of DCs^11^. Myeloid-derived suppressor cells (MDSCs) have emerged as important contributors to tumor progression and have been implicated in limiting the effects of cancer immunotherapy. MDSCs consist of two subgroups of cells, polymorphonuclear MDSCs, which are characteristically similar to neutrophils, and monocytic MDSCs, which are phenotypically and morphologically similar to monocytes. AK036396 and RNCR3 regulate MDSC maturation and differentiation^11^. Therefore, lncRNAs are expressed in different types of immune cells and have roles in regulating cancer immunity by modulating immune cell functions. This characteristic should be considered during the development of lncRNA-targeted therapies.

**Table 2:** Overview of lncRNA role in regulating innate cells in the TIME

LncRNA	Cancer types	Immune cells	Regulated targets	Mechanisms	Reference
Lnc-MC	—	Macrophage	miR-199a-5p/ACVR1B	Lnc-MC participates in macrophage differentiation *via* regulating miR-199a-5p/ACVR1B axis	^12^
GNAS-AS1	BC	Macrophage	miR-433/GATA3	miR-433/GATA3 axis promotes M2 macrophage polarization	^12^
LINC00662	HCC	Macrophage	Wnt/β-catenin	LINC00662 activates Wnt/β-catenin signaling to promote M2 macrophage polarization	^12^
MALAT1	HCC	Macrophage	miR-140/VEGF	MALAT1 inhibits M1 macrophage polarization *via* regulating miR-140/VEGF axis	^12^
ANCR	GC	Macrophage	FoxO1	ANCR promotes FoxO1 degradation, thereby inhibits M1 macrophage polarization	^12^
XIST	BC	Macrophage	miR-503	XIST increases the secretion of exosomes miR-503, which can induce M1/M2 polarization	^12^
CamK-A	BC	Macrophage	NF-κB	CamK-A activates NF-κB, then promotes macrophage recruitment	^12^
Lnc01116	Glioma	Neutrophil	IL-1β	Lnc01116 promotes neutrophil recruitment through increasing IL-1β expression	^12^
Lnc-CD56	—	NKs	CD56	Lnc-CD56 promotes NKs maturation *via* increasing CD56 expression	^12^
Lnc00657	CC	NKs	miR-20a-5p/DR5	Lnc00657 enhances the cytotoxicity of NKs *via* regulating miR-20a-5p/DR5 axis	^12^
LncGAS5	HCC	NKs	miR-544/RUNX3	LncGAS5 induces NKs to secrete IFN-γ through regulating miR-544/RUNX3 axis	^12^
Lnc-DC	—	DCs	STAT3	Lnc-DC interacts with STAT3 to promote DCs differentiation	^12^
HOTAIRM1	—	DCs	miR-3960	HOTAIRM1 suppresses DCs differentiation *via* binding to miR-3960	^12^
Lnc-Dpf3	—	DCs	HIF-1α	Lnc-Dpf3 inhibits DCs migration *via* inhibiting HIF-1α mediated Ldha expression	^12^
AK036396	—	MDSCs	Fcnb	AK036396 inhibits the maturation of MDSCs by enhancing Fcnb stability	^12^
RNCR3	—	MDSCs	miR-185-5p/Chop	RNCR3 acts as a competitive RNA to promote the differentiation of MDSCs *via* the miR-185-5p/Chop pathway	^12^
MIR4435-2HG	CRC	Neutrophil	BIM	MIR4435-2HG inhibits BIM expression, resulting in decreased neutrophil apoptosis	^13^

BC, breast cancer; HCC, hepatocellular carcinoma; GC, gastric cancer; CC, cervical cancer; CRC, colorectal cancer; NKs, natural killer cells; DCs, dendritic cells; MDSCs, myeloid-derived suppressor cells.

## LncRNAs regulate adaptive immune cells in the TIME

**Figure 3:**
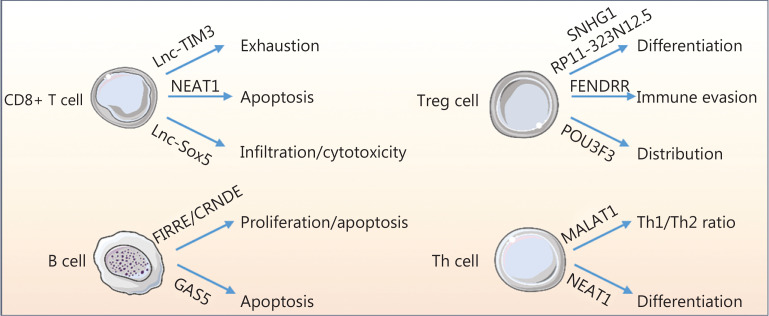
LncRNAs regulate adaptive immune cells. LncRNAs mediate CD8+ T cell exhaustion, apoptosis, infiltration, and cytotoxicity. In addition, lncRNAs control the ratio of Th1-to-Th2 cells and regulate Th cell differentiation. Furthermore, lncRNAs mediate the differentiation, distribution, and immune evasion of Tregs. Additionally, lncRNAs regulate B-cell proliferation and apoptosis.

The adaptive immune system, including T and B cells, is responsible for initiating anti-tumor immune responses and immunologic memory^2^. T cells are the major immune cells involved in adaptive immunity^2^ and are classified into multiple subgroups based on immune functions and immunomodulatory roles. Importantly, the diverse subtypes of T cells in the TME have a strong correlation with the prognostic significance of PD-1/PD-L1 blockade therapy. Recently, lncRNAs have been shown to be functionally relevant in adaptive immunity^2^. A rapidly accumulating body of evidence suggests that lncRNAs from cancer or immune cells in the TME have many decisive and indispensable functions in T and B cell viability, migration, proliferation, and cytotoxicity^14^ (**Table 3**, **Figure 3**). CD8+ T cells are the major tumor-killing effector T cells in the tumor bed. Numerous studies over the last few years have revealed that lncRNAs, such as *NEAT1* and *lnc-TIM3*, have crucial roles in CD8+ T cell apoptosis and exhaustion^14^. In addition to viability and migration, CD8+ T cell cytotoxic function determines the anti-tumor effects. *Lnc-Sox5* promotes tumor immune evasion by inhibiting CD8+ T cell infiltration and cytotoxicity^14^. T helper cells are critical for activation of the anti-tumor response alone of *via* stimulation of CD8+ T cells. The lncRNAs *MALAT1* and *NEAT1*, participate in the differentiation of naive T helper cells into specialized populations of effector cells, including anti-tumorigenic Th1, pro-tumorigenic Th2, and Th17 cells^11^. Regulatory T cells (Tregs) comprise another pro-tumorigenic T helper cell population. The differentiation and distribution of Tregs regulate pro-tumorigenic functions. LncRNAs (*SNHG1*, *POU3F3*, and *RP11-323N12.5*) regulate cancer cell immune evasion by mediating the differentiation and distribution of Tregs^14^. The adaptive immune system also relies on B cell function. In recent years, the role of T cells in anti-tumor immunity has been extensively studied; however, research on B cells is still in its infancy. B cells have been shown to differentiate into plasma cells after stimulation with tumor antigens. Plasma cells then migrate to the tumor site and secrete various cytokines and antibodies to amplify the anti-tumor immune response *via* phagocytosis and complement activation. LncRNAs, such as *FIRRE*, *GAS5*, and *CRNDE*, affect B cell proliferation and apoptosis *via* the regulation of gene expression^11^. In fact, approximately 30% of coding genes are transcribed during B cell differentiation; however, the number of aberrant lncRNAs is far greater than the number of coding genes. This finding emphasizes the importance of lncRNAs in B cell function.

**Table 3:** Overview of the lncRNA role in regulating adaptive cells in the TIME

LncRNA	Cancer types	Immune cells	Regulated targets	Mechanisms	Reference
MALAT1	LAD	Th1/Th2	miR-155	MALAT1 interacts with miR-155 to increase the Th1:Th2 ratio	^12^
NEAT1	—	Th17 cells	STAT3	NEAT1 inhibits STAT3 expression to suppress CD4+ T cell differentiation into Th17 cells	^12^
FIRRE	DLBCL	B cells	Wnt/β-Catenin	FIRRE promotes DLBCL proliferation by regulating the Wnt/β-catenin signaling pathway	^12^
GAS5	DLBCL	B cells	miR-222	GAS5 interacts with miR-222 to promote DLBCL apoptosis	^12^
CRNDE	BCP-ALL	B cells	miR-345-5p	CRNDE reduces BCP-ALL apoptosis *via* sponging miR-345-5p	^12^
FENDRR	HCC	Treg	GADD45B	FENDRR increases GADD45B expression to inhibit Treg-mediated immune escape	^15^
SNHG1	BC	Treg	miR-448/IDO	SNHG1 contributes to Treg differentiation and promotes cancer cell immune evasion *via* the miR-448/IDO signaling pathway	^15^
POU3F3	GC	Treg	TGF-β	POU3F3 promotes Treg distribution by activating TGF-β signaling pathway	^15^
RP11-323N12.5	GC	Treg	YAP	RP11-323N12.5 induces Treg differentiation by enhancing YAP transcription	^15^
NEAT1	HCC	CD8+T cells	miR-155/Tim-3	NEAT1 enhances CD8+T cell apoptosis by regulating the miR-155/Tim-3 signaling pathway	^15^
Lnc-TIM3	HCC	CD8+T cells	Tim-3	Lnc-TIM3 interacts with Tim3 to suppress NFAT1 and AP-1, thus leading to CD8+ T cell exhaustion	^15^
Lnc-Sox5	CRC	CD8+T cells	IDO1	Lnc-Sox5 promotes IDO1 expression to inhibit CD8+ T cell infiltration	^15^

HCC, hepatocellular carcinoma; CRC, colorectal cancer; LAD, lung adenocarcinoma; BC, breast cancer; GC, gastric cancer; DLBCL, diffuse large B cell lymphoma; BCP-ALL, B-cell precursor acute lymphoblastic leukemia; Th1, T helper type 1; Th2, T helper type 2; Th17, T helper type 17; Treg, T regulatory cell.

## RNA therapeutic prospects

As described above, lncRNAs expressed in cancer or immune cells contribute to immune evasion. LncRNAs represent promising molecules that may serve as prognostic biomarkers or potential therapeutic targets in combination with anti-PD-1/PD-L1 antibodies. Over the past decade, substantial efforts have been made towards the clinical application of RNA-based therapeutics. Approval of an mRNA-based vaccine against SARS-CoV-2 demonstrated the clinical feasibility, safety, and efficacy of such therapeutics^16^. These findings have paved the way for the application of RNA therapeutics in cancer immunotherapy. Initially, RNA was considered unsuitable as a therapeutic target because RNA is resistant to degradation by ribonucleases^17^. In addition, conventional RNA agents may induce toxicity and immune responses, thereby limiting the development of RNA-based treatment strategies^17^. Many shortcomings have been identified with these treatment strategies, including the chemical modification of the RNA structure in parallel with RNA delivery techniques and protection technology applications^18^. Compared to conventional drug formats, RNA therapeutics has a regulatory role in cancer progression, mainly by controlling the levels of target protein expression. The most prevalent RNA therapeutics can be divided into two main groups: coding RNA *in vitro* transcribed-messenger RNA (iVT-mRNA); and self-amplifying RNA (SAM) and non-coding RNAs (ncRNAs), including lncRNA, circular RNA (circRNA), miRNA, small interfering RNA (siRNA), and antisense oligonucleotides (ASOs)^16^. Although these ncRNAs cannot be translated into proteins to perform oncogenic or suppressor functions as can coding RNAs, ncRNAs participate in cancer cell biology to varying degrees. Hence, ncRNA-based approaches are an attractive field in cancer medicine research and provide a rationale for clinical application in cancer treatment. In particular, because RNA therapeutics have immunomodulatory functions, RNA molecules are highly desirable for immunotherapy^19^.

## LncRNA-based cancer immunotherapy

**Figure 4:**
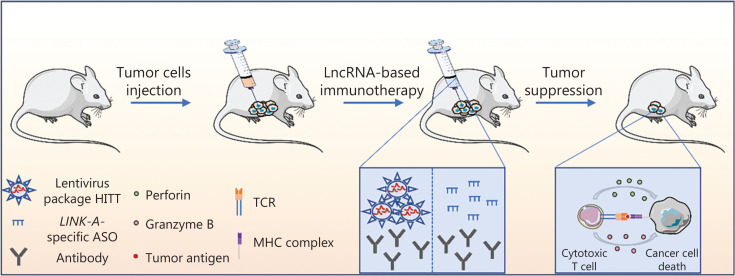
LncRNA-based cancer immunotherapy prospects. The *in situ* injection of ICB combined with HITT expression lentivirus particles or the combination of an immune checkpoint inhibitor with a *LINK-A*-specific ASO produced an obvious anti-tumor effect by promoting the tumor cell killing activity of cytotoxic T cells.

Coding RNA-based therapies, represented by mRNA therapeutics, have emerged as powerful tools for the treatment of various human diseases, especially malignant tumors. By targeting multiple tumor-specific neoantigens or tumor suppressor genes and by introducing mRNA-based cancer vaccines, mRNA-based treatments have been shown to elicit passive anti-tumor immune responses by activating the innate and adaptive immune systems. NcRNAs constitute > 90% of the RNAs in the human genome; however, most of the > 50,000 known ncRNAs have only been discovered in the past 10 years. Some of the ncRNAs have been shown to have essential roles in modulating the TIME. SiRNAs and miRNAs bind to targets *via* base-complementary binding to effectively and specifically regulate target gene expression. These RNAs can affect CD8+ T cells, Treg infiltration, macrophage differentiation, and DC presentation. More recently, ASO-based therapeutic strategies have been characterized by targeting non-degradable RNAs using other RNA silencing approaches. Many ASOs have shown promising immunomodulatory roles, such as regulating T cell differentiation, macrophage polarization, and MDSC polarization, by manipulating the expression of their target genes. CircRNAs have been reported to sponge miRNA to regulate MDSC migration and NK and CD8+ T cell cytotoxic activity in the TME. Although the findings between tumor and immune cells indicate the potential of lncRNAs as advanced therapeutic targets, the combination of immunotherapy and lncRNA-based targeted therapies is still in its infancy. The reason for this situation may be multifaceted, such as the length of lncRNAs being > 200 bp, which is not suitable for RNA delivery systems. In addition, RNAs > 200 bp in length are more susceptible to degradation by ubiquitous ribonucleases. Therefore, further narrowing down the functional lncRNA domain sequence with the results of basic research will contribute to promote the feasibility of lncRNA-based immunotherapy. Recently, an anti-tumor and pro-immunity lncRNA, HIF-1α inhibitor at translation level (*HITT*), was identified^20^. *HITT* coordinates with regulator of G protein signaling 2 (RGS2) to bind to the 5′-untranslated region of *PD-L1*, which leads to reduced PD-L1 translation and the subsequent activation of CD8+ T cells^20^. The functional fragment in *HITT* was narrowed down from 2,000 nt to 62 nt (1,080–1,142 nt). Notably, third-generation lentivirus packaging systems have been approved for the treatment of human diseases owing to the high levels of safety and efficiency^21^. Interestingly, a significant anti-tumor immunotherapy effect was achieved by the intra-tumor injection of the lentivirus expressing the functional sequence of *HITT*. The combination of anti-PD-1 therapy and lentivirus particles expressing the *HITT* fragment produced a synergistic anti-tumor effect when compared to monotherapy with an anti-PD-1 antibody^20^ (**Figure 4**). In addition, preclinical studies have shown that lncRNAs, which play roles in modulating cancer immunity through diverse mechanisms, such as reducing antigen presentation or MHC molecule expression, can be used as potential targets to improve ICB efficiency. For example, Hu et al.^22^ showed that ASOs specifically targeting *LINK-A*, an immunosuppressive lncRNA, are able to inhibit *LINK-A* expression and sensitize tumor cells to ICB therapy by increasing tumor cell MHC expression (**Figure 4**). These data indicated the potential application of lncRNAs in the treatment of cancer by boosting T cell immunity or enhancing the efficiency of PD-1/PD-L1 blockade.
